# Emergence of Colistin Resistance in Carbapenem-Resistant Hypervirulent *Klebsiella pneumoniae* Under the Pressure of Tigecycline

**DOI:** 10.3389/fmicb.2021.756580

**Published:** 2021-12-01

**Authors:** Yawei Zhang, Xiaojuan Wang, Shifu Wang, Shijun Sun, Henan Li, Hongbin Chen, Qi Wang, Hui Wang

**Affiliations:** ^1^Department of Clinical Laboratory, Peking University People’s Hospital, Beijing, China; ^2^Department of Clinical Microbiology, Qilu Children’s Hospital of Shandong University, Jinan, China

**Keywords:** hypervirulent *Klebsiella pneumonia*, colistin resistance, experimental evolution, virulence, fitness

## Abstract

Colistin and tigecycline are the last options against carbapenem-resistant hypervirulent *Klebsiella pneumoniae* (CR-hvKP). Intersecting resistance determinants have been detected between these antibiotics; however, there is only limited evidence of such association. Here, we describe a colistin-resistant CR-hvKP isolated from a patient with severe neonatal bacteremia treated with tigecycline as opposed to colistin before isolation of this strain, providing a clinical clue to colistin resistance under tigecycline pressure. Furthermore, an ST11-K64 KPC-2–producing, colistin-susceptible CR-hvKP strain was subjected to experimental evolution toward colistin resistance under tigecycline and colistin pressure to verify this phenomenon *in vitro*. The biological impact of acquiring colistin resistance on fitness and virulence was also studied. As expected, the parental strain rapidly developed colistin resistance under both tigecycline and colistin selection. However, different from the colistin resistance mechanism in the clinical strain that was due to an IS*Kpn26* insertion in the *mgrB* gene, the mutants in this study developed colistin resistance through a ∼4.4 or ∼4.6 kb deletion including the *mgrB* locus as well as the *kdgR*, *yobH*, *yebO*, *yobF*, *cspC*, *ftsI*, and *rlmA* genes. Although the virulence of the colistin-resistant mutants, as determined in the *Galleria mellonella* model, decreased compared with that of the parent strain, it was still higher than that of NTUH-K2044. This suggests a slight virulence cost when CR-hvKP develops colistin resistance under tigecycline or colistin pressure. Together, our results provide clinical and experimental evidence for the association between colistin resistance and tigecycline pressure in CR-hvKP, highlighting a critical issue in the clinical setting.

## Introduction

Carbapenem-resistant *Klebsiella pneumoniae* (CRKP) is a critical threat to public health (Van Duin and Doi, 2017). Globally, the prevalence of CRKP has markedly increased. In China, the meropenem resistance rate in *K. pneumoniae* has increased from 2.9% in 2005 to 24.2% in 2020 (CHINET, 2021). *K. pneumoniae* carbapenemase (KPC)–2–producing ST11 clones of CRKP are of particular concern because they can spread rapidly and cause severe infections in clinical settings (Fraenkel-Wandel et al., 2016; Wang et al., 2018). In recent years, we have witnessed the emergence of carbapenem-resistant hypervirulent *K. pneumoniae* (CR-hvKP). Notably, the virulence increased in the KPC–2–producing ST11 clones in mainland China, from 2.1 to 7.0% between 2015 and 2017 (Zhang et al., 2020). In addition, more than 80% of patients with respiratory tract and bloodstream infections caused by CR-hvKP experience poor outcomes (Gu et al., 2018; Mohammad Ali Tabrizi et al., 2018).

The threat of CR-hvKP is substantial, as few therapeutic options remain. Colistin is considered a last-line antibiotic for treating CR-hvKP infections. Previous studies have demonstrated that colistin has excellent *in vitro* activity against CR-hvKP strains (Yu et al., 2018; Hao et al., 2020; Lin et al., 2020). However, cases of colistin-resistant CR-hvKP are increasing in clinical practice in China (Dong et al., 2018; Huang et al., 2018; Solgi et al., 2020; Jin et al., 2021). The common mechanisms mediating colistin resistance in *K. pneumoniae* include chromosomal mutation of the genes associated with the modification of lipid A, such as those encoding the enzymes involved in lipid A synthesis and the PmrAB and PhoPQ two-component systems (Hamel et al., 2021). Other resistance strategies include the efflux pump mechanism (AcrAB–TolC and SoxSR), which is similar to many of the described tigecycline resistance mechanisms (Hamel et al., 2021). Given these data, we speculated that there may be a close association between colistin and tigecycline resistance in CR-hvKP strains. We previously searched the PubMed database using the terms “colistin” AND “carbapenem-resistant hypervirulent *K. pneumoniae*” for articles published up to July 1, 2021, with no language restrictions, and retrieved five papers describing colistin-resistant CR-hvKP infections. These reports were based on case studies and did not evaluate the potential fitness cost of acquiring colistin resistance in these strains of CR-hvKP.

Herein, we obtained a KPC–2–producing CR-hvKP strain, C2582, which belongs to the ST11-K64 clones from the China Carbapenem-Resistant Enterobacteriaceae (CRE) Network. This strain demonstrated colistin resistance and hypervirulence in the *Galleria mellonella* infection model (Zhang et al., 2020). We investigated its clinical background, antibiotic exposure, colistin resistance mechanism, and genomic features. We further elucidated the potential evolutionary mechanisms of colistin resistance and change in fitness and virulence in response to acquired colistin resistance among the CR-hvKP strains. Our study provides evidence for the link between colistin resistance and tigecycline pressure.

## Materials and Methods

### Bacterial Strains and Clinical Information

The background information for colistin-resistant CR-hvKP C2582 can be found in our previous study (Zhang et al., 2020). C2582 belonged to the ST11–K64 clone and was resistant to carbapenems (MIC > 32 mg/L) and colistin (MIC of 8 mg/L) but susceptible to tigecycline (MIC of 1 mg/L). Clinical information, including demographic data, underlying diseases, specimen type, isolation date, clinical manifestations, antibiotic exposure, invasive device usage, and outcomes, was collected and reviewed.

### Antimicrobial Susceptibility Testing

Both the agar and broth microdilution methods for antimicrobial susceptibility were performed according to the guidelines established by the Clinical and Laboratory Standards Institute (CLSI), and the data were interpreted using the Food and Drug Administration and CLSI breakpoints for tigecycline and other antimicrobials, respectively (Food and Drug Administration [FDA], 2009; Clinical and Laboratory Standards Institute [CLSI], 2015, 2020). *Pseudomonas aeruginosa* ATCC 27853 and *Escherichia coli* ATCC 25922 were used as controls.

### *In vitro* Experimental Evolution of Colistin Resistance in CR-hvKP

The neonatal patient in this study was exposed to tigecycline rather than colistin before we isolated colistin-resistant CR-hvKP strain C2582 from the blood sample. Thus, we selected a single CR-hvKP isolate for the *in vitro* evolution of acquired colistin resistance under both tigecycline and colistin pressures. An ST11–K64 KPC-2–producing colistin-susceptible CR-hvKP strain (C1398), which was isolated in our previous study and phylogenetically close to C2582, was subjected to an experimental evolution assay designed to produce colistin resistance using passage on increasing concentrations of tigecycline or colistin over a five-day period, as previously described (Rodriguez de Evgrafov et al., 2015; Zhang et al., 2020). Briefly, 100 μL of the diluted (1:100) overnight culture of C1398 was added to 100 μL of Mueller–Hinton broth supplemented with a gradient concentration of colistin or tigecycline (from 0.064 to 64 mg/L). After overnight incubation, the culture from the highest concentration of colistin or tigecycline that had visible growth was diluted and transferred to the next stage. Resistant mutants from each cultivation were frozen in glycerol at –80°C before being used in the antimicrobial susceptibility testing. Each antibiotic was evaluated using two biological replicates. The mutants from the fifth day of colistin selection are called C1398 C-1 and C1398 C-2, while the mutants from the tigecycline selection are called C1398 T-1 and C1398 T-2.

### *Galleria mellonella* Infection Model

A total of 20 pathogen-free *G. mellonella* larvae (Huiyude Biotech Company, Tianjin, China) weighing 250–350 mg were used to determine the *in vivo* virulence of our mutant strains (Insua et al., 2013). A mid-log phase culture with an optical density at 600 nm (OD_600_) of 0.6, was washed and diluted in phosphate-buffered saline (PBS) to produce a solution with 10^7^ cfu/mL. Each larva was injected in their left proleg with 10 μL of bacterial suspension using a microsample syringe, and groups injected with PBS, ZR2, and NTUH-K2044 were used as the blank, negative, and positive control groups, respectively. After injection, larvae were placed in Petri dishes at 37°C in the dark. Mortality rates were then evaluated at 72 h.

### Growth Curve Assay

To evaluate the association between colistin resistance and fitness, the growth curves of the parent strain, C1398, and its corresponding mutants were compared as described previously (Zhang et al., 2020). Bacteria were inoculated in 5 mL of LB broth at 37°C with shaking (200 rpm) until the mid-log phase, before being diluted to an OD_600_ of 0.01 with LB broth. A total of 200 μL from each sample was then transferred to a 96-well microtiter plate, and the OD_600_ value was recorded every 30 min for 24 h. Each experiment was completed in triplicate.

### Whole-Genome Sequencing

Clinical colistin-resistant CR-hvKP strain, C2582, was sequenced using the Illumina HiSeq and PacBio RS II systems (Pacific Biosciences), and these data were used to characterize its plasmids. The day five mutants from the C1398 experiments were also sequenced using Illumina HiSeq (Illumina, San Diego, CA, United States) at 100 × coverage, and Oxford Nanopore GridlON X5 instrument (Oxford Nanopore Technologies). The Unicycler pipeline v0.4.7 and SPAdes v3.13.0 were used for *de novo* assembly, and the draft genome sequence was annotated using Prokka and Rapid Annotation Subsequencing Technology (RAST).^1^ Resistance genes, serotypes, plasmid replicon types, virulence genes, and IS elements were analyzed by the Center for Genomic Epidemiology,^2^ Kleborate,^3^ and ISFinder, whereas a BLAST Ring Image Generator (BRIG) was used to visualize the plasmid. We also compared single nucleotide polymorphisms (SNPs) in the parent strain C1398 (accession no. CP034420-CP034424) and its corresponding mutants using Burrows-Wheeler Aligner, Picard tools, Genome Analysis Tool Kit (GATK), and BLAST. The complete sequences for C2582 and the mutants of C1398 were deposited in the GenBank database under accession no. CP079208-CP079211 and CP080584-CP080587, respectively.

### Efflux Pump Inhibitory Assays

PAβN and CCCP inhibitory tests were used to explore the role of efflux pumps in both the colistin- and tigecycline-resistant strains (Ni et al., 2016). MIC values for colistin and tigecycline (Pfizer, NY, United States) were determined using the broth microdilution method as recommended by the CLSI guidelines (Clinical and Laboratory Standards Institute [CLSI], 2015). Briefly, stock solutions of PAβN (Sigma-Aldrich, Shanghai, China) and CCCP (Sigma-Aldrich, Shanghai, China) were prepared at 10 mg/mL in sterile water and DMSO, respectively. Bacterial growth in Mueller-Hinton broth containing tigecycline or colistin with and without PAβN (25 mg/L) or CCCP (10 mg/L) was then evaluated in parallel. A growth control with 25 mg/L PAβN or 10 mg/L CCCP in Mueller-Hinton broth was added to evaluate the effect of each of the efflux pump inhibitors on their own in each strain.

### Real-Time Quantitative Reverse Transcription PCR (RT-qPCR)

The expression levels of various colistin-resistance-associated genes (*phoP*, *pmrD*), efflux pump-encoding genes (*acrB*, *tolC*), and the global *ramA* regulon were examined by RT-qPCR. Overnight cultures of the parent strain C1398 and its corresponding mutants were inoculated into LB broth and incubated at 37°C for 4 h with shaking (200 rpm). The mid-log phase culture was harvested, and RNA was extracted using the RNeasy midi kit (Qiagen Sciences, Germantown) with DNase I treatment to remove DNA contamination. cDNA was then produced from 1,000 ng of total RNA using the PrimeScript RT reagent kit (Takara). RT-qPCR was performed using a Roche Cobas z 480 real-time PCR analyzer (Roche Diagnostics Ltd, Mannheim, Germany). Each RT-qPCR sample included the same RNA preparations. *The rrsE* gene was used as an internal control for quantifying relative gene expression. Each strain was tested three times.

### Statistical Analysis

All statistical tests were performed using one-way analysis of variance (ANOVA) or Student’s *t*-test (Prism v5 software). Survival curves were assessed by Kaplan-Meier analysis and log-rank test. Statistical significance was set at *p* < 0.05.

### Ethical

This study was approved by the Ethics Committee of Peking University People’s Hospital (reference number 2019PHB194-01). Written informed consent was waived for the patient, as this research was retrospective and the patient’s data were anonymized.

## Results and Discussion

### Clinical Case of Colistin-Resistant CR-hvKP

**FIGURE 1:**
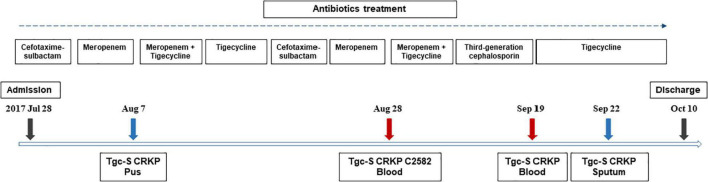
Timeline showing antimicrobial exposure and bacterial culture results in the infant patient infected with colistin-resistant CR-hvKP strain C2582. Strains other than C2582 were not acquired in this study.

On July 28, 2017, a 3-month-old female infant with a history of choledochal cyst surgeries presented with fever (39.9°C), diarrhea, and hematochezia and was admitted to the hospital before being diagnosed with intestinal obstruction, volvulus, intestinal necrosis, intestinal perforation, and enteritis. Cefotaxime-sulbactam and later, meropenem, were used to treat the infections. Intestinal resection and fistula were performed during hospitalization. Pus cultures from the lesion were shown to be tigecycline-susceptible carbapenem-resistant *K. pneumoniae* (CRKP), and the patient was then treated with a combination of tigecycline and meropenem. After stabilizing the patient, this treatment was changed to cefotaxime/sulbactam. However, the patient subsequently presented with fever, and a series of tigecycline-susceptible CRKP strains were successively isolated from her blood and sputum samples, one of which, C2582, was recovered from a blood sample. Her treatment was then changed to tigecycline and meropenem. Subsequent clinical improvement meant that the patient was discharged after 75 days in the hospital. The timeline describing the antimicrobial exposure and bacterial culture results from patients infected with colistin-resistant CR-hvKP strain C2582 are summarized in Figure 1.

### Genomic Features of C2582

**FIGURE 2:**
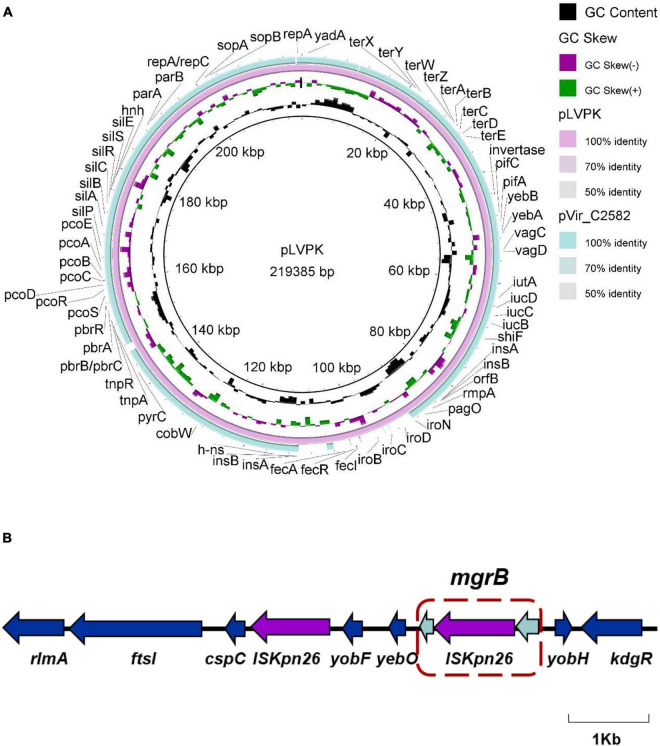
Comparative analysis of the virulence plasmid of C2582. **(A)** Alignment analysis of virulence plasmid sequences between pVir_C2582 and previously reported virulence plasmid pLVPK (accession no. AY378100). **(B)** Schematic representation of the insertion events that occurred in the *mgrB* gene.

We obtained the whole-genome sequence of C2582 using hybrid *de novo* assembly of both short-and long-read sequencing data. The complete genome was assembled in four contigs, one chromosome (5,514,392–bp), one virulence plasmid (216,679–bp, designated pVir-C2582) carrying *iucABCD*, *rmpA*, and *rmpA2*, one IncFII multi-drug resistance plasmid (128,299–bp, designated pRes-C2582) harboring *bla*_KPC–2_, and one IncFIB plasmid (110,121 bp). Several genes mediating β-lactam (*bla*_SHV–12_, *bla*_CTX–M–65_, *bla*_TEM–1B_), aminoglycoside (*rmtB*), and fosfomycin (*fosA3*) resistance were also found on the resistance plasmid, pRes-C2582. In the case of the KPC plasmids, pRes-C2582 shared high similarity with the *bla*_KPC–2_-encoding plasmid described in the GenBank database, with more than 99% identity and 100% coverage. C2582 also carried a pLVPK-like virulence plasmid, sharing 92% coverage and 99% identity with previously described virulence plasmid pLVPK (accession no. AY378100) (Figure 2A). Unlike other hypervirulent *K. pneumoniae*, which usually carry an IncHI1B/IncFIB virulence plasmid, C2582’s virulence plasmid was shown to belong to the IncHI1B replicon type, and we assumed that the IncHI1B/IncFIB pLVPK-like plasmid decomposed in an effort to adapt to antibiotic pressure.

We also evaluated our sequences for mutations in the *mgrB*, *phoPQ*, *pmrAB*, *pmrCD*, and *pmrH* genes, all of which are associated with various mechanisms for acquired colistin resistance. We identified an IS*Kpn26* insertion at the 75 nucleotide (nt) position of *mgrB* using the WGS data and confirmed this by PCR (Figure 2B and Table 1). We previously searched the PubMed database for reports on colistin-resistant CR-hvKP, and to the best of our knowledge, only five papers describing colistin-resistant CR-hvKP had been published. In addition, the majority of instances of reported colistin-resistance were attributable to changes in the *mgrB* gene, including IS element mutations such as the insertion of IS*Kpn18* or IS*Kpn26* (Dong et al., 2018; Huang et al., 2018; Solgi et al., 2020) and chromosomal mutations such as Q30stop and C39F (Shelenkov et al., 2020; Jin et al., 2021).

**TABLE 1:** Antimicrobial susceptibility profiles and the resistance determinants of the clinical CR-hvKP strain C2582, parent CR-hvKP strain C1398, and the corresponding mutants of C1398 selected by colistin and tigecycline on the fifth day.

Antimicrobials	Clinical strain C2582 (mg/L)	Parent strain C1398 (mg/L)	Mutants selected by tigecycline (mg/L)		Mutants selected by colistin (mg/L)	
			C1398 T-1	C1398 T-2	C1398 C-1	C1398 C-2
Cefotaxime	>128	>128	> 128	>128	>128	>128
Ceftazidime	>64	64	64	64	64	64
Cefepime	128	128	128	128	128	128
Meropenem	>32	64	128	128	128	128
Piperacillin-tazobactam	>256	>256	>256	>256	>256	>256
Amikacin	>256	1	0.5	0.5	0.5	1
Tigecycline	1	0.5	2	4	1	0.5
Colistin	8	≤ 0.125	64	≤0.125	16	128
Element for tigecycline MIC change	–	–	*lon* [1,286–1,295 nt deletion (CTTCCGACAT)]	*ramR* [206–217 nt deletion (CAATGATCGCCG)]	*ramR* [207–213 nt deletion (AATGATC)]	–
Element for colistin MIC change	*mgrB* (75nt IS*Kpn26*–like insertion)	–	*mgrB* (4,455–bp deletion, including *kdgR*, *yobH*, *mgrB*, *yebO*, *yobF*, *cspC*, *ftsI*, and *rlmA*)	–	–	*mgrB* (4,625–bp deletion, including *kdgR*, *yobH*, *mgrB*, *yebO*, *yobF*, *cspC*, *ftsI*, and *rlmA*)
Increased expression levels in the genes	–	–	*pmrD*, *acrB*, *tolC*, *ramA*	*pmrD*, *acrB*, *tolC*, *ramA*	*acrB*, *tolC*, *ramA*	*phoP*, *pmrD*

### CR-hvKP Rapidly Developed Colistin Resistance

Recent studies have shown that efflux pumps such as AcrAB-TolC and SoxSR are required for colistin resistance in *K. pneumoniae* (Hamel et al., 2021), suggesting that they use a similar mechanism of resistance to that of the tigecycline-resistant strains. Furthermore, the neonatal patient in this study was not treated with colistin, but rather with tigecycline before the colistin-resistant CR-hvKP strain was isolated. Therefore, we speculated that the antibiotic pressure from the tigecycline treatment may induce colistin resistance in CR-hvKP, which prompted us to study the experimental evolution of colistin resistance in response to both tigecycline and colistin pressure and analyze the potential risk and biological impacts of colistin resistance in these strains. A single ST11-K64 KPC-2–producing colistin-susceptible CR-hvKP strain, C1398, was selected as the parental strain in this study.

**FIGURE 3:**
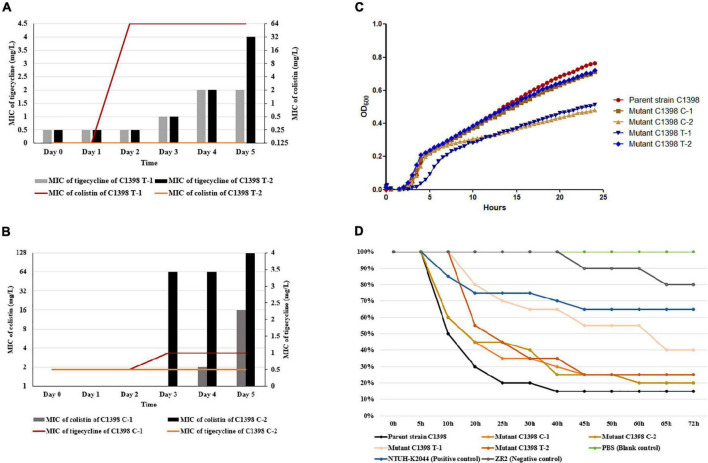
Experimental acquisition of colistin resistance and its biological effects on CR-hvKP. **(A)** Experimental evolution of colistin resistance under tigecycline pressure. **(B)** Experimental evolution of colistin resistance under colistin pressure. **(C)** Growth curve for parental strain C1398 and its mutants. Similar kinetics were observed for the parent strain, C1398, and mutants C1398 C-1 (*p* = 0.639) and C1398 T-2 (*p* = 0.8551), while significant differences in growth kinetics were observed for C1398 C-2 (*p* = 0.0028) and C1398 T-1 (*p* = 0.0017) when compared to the parental control. Data are presented as the mean ± standard deviation of three independent experiments. **(D)** Changes in the *in vivo* virulence of these resistant strains as evidenced by the *Galleria mellonella larvae* infection model. *G. mellonella* mortality was evaluated over a 72 h period.

As expected, the parental strain rapidly developed resistance to colistin (C1398 T-1), with this mutant appearing on the second day of culture with tigecycline. The second mutant selected under tigecycline (C1398 T-2) was not susceptible to tigecycline (MIC of 4 mg/L) but was susceptible to colistin (MIC ≤ 0.125 mg/L), indicating that these isolates used different evolutionary pathways to develop resistance (Figure 3A). The MIC for colistin increased more rapidly than that of tigecycline under tigecycline selection in C1398 T-1 (Figure 3A). Meanwhile, the parent strain, C1398, produced colistin-resistant mutants after 3 days of colistin pressure (Figure 3B). The antimicrobial susceptibility profiles and resistance determinants of each of the mutants produced using colistin and tigecycline selection (designated mutants C1398 C-1, C1398 C-2, C1398 T-1, and C1398 T-2, respectively) are listed in Table 1.

To determine the impact of acquiring colistin resistance on both the virulence and fitness of these CR-hvKP isolates, we evaluated their growth kinetics and virulence using growth curves and the *G. mellonella* infection assay. The parent strain, C1398, and colistin-resistant mutant, C1398 C-1, presented with fairly similar growth kinetics (*p* = 0.639), while there were significant differences in the growth rate of C1398 and the other colistin-resistant mutants, C1398 C-2 (*p* = 0.0028) and C1398 T-1 (*p* = 0.0017) (Figure 3C). In addition, similar virulence was observed for the parent strain and the colistin-resistant mutants C1398 C-1 (*p* = 0.443) and C1398 C-2 (*p* = 0.385), while significant differences in virulence were shown for C1398 T-1 (*p* = 0.003) and C1398 T-2 (*p* = 0.043) when compared to the parental control (Figure 3D). Although the virulence of the colistin-resistant mutant selected under tigecycline, C1398 T-1, was lower than that of the parent strain, it was still higher than that of the positive control, NTUH-K2044 (Figure 3D), suggesting a potential risk and limited virulence cost for CR-hvKP when developing colistin resistance under tigecycline or colistin pressure.

### Colistin Resistance Emerges via Different Evolutionary Pathways in CR-hvKP Strains

**FIGURE 4:**
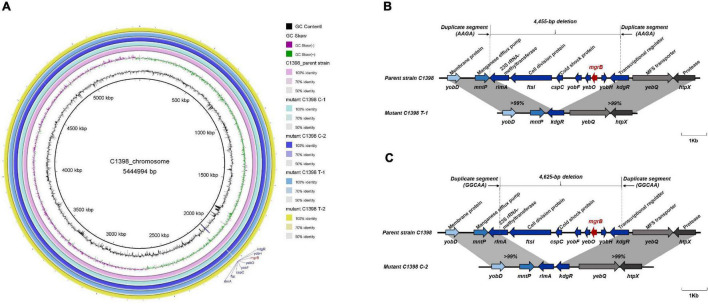
Comparative analysis of the chromosome sequences among the parental strain C1398 and its mutants. The mutants from the fifth day of colistin selection are called C1398 C-1 and C1398 C-2, while the mutants from the tigecycline selection are called C1398 T-1 and C1398 T-2. **(A)** Alignment analysis of chromosome sequences of the parental strain C1398 and its corresponding mutants. **(B,C)** A ∼4.4 and 4.6 kb deletion containing *mgrB* locus and several other genes, including the transcriptional regulator *kdgR*, *yobH*, *yebO*, *yobF*, cold shock protein gene *cspC*, cell division protein gene *ftsI*, and *rlmA* were identified in mutant C1398 T-1 and mutant C1398 C-2, respectively.

**FIGURE 5:**
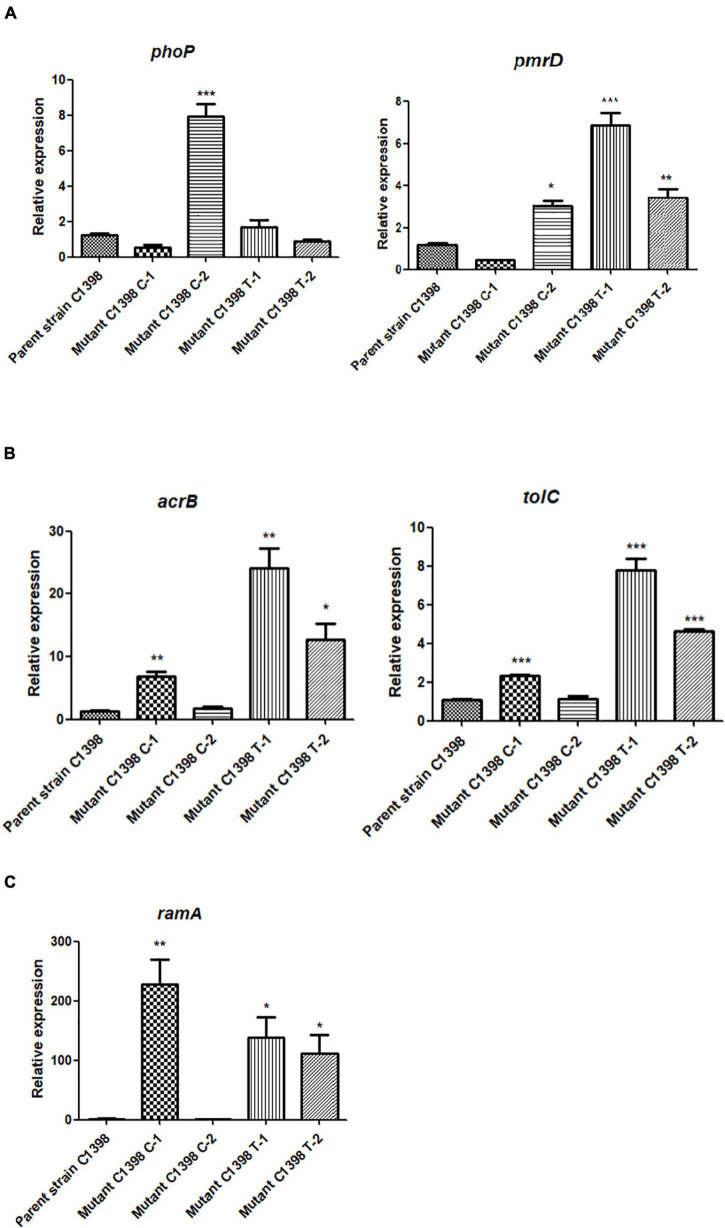
Relative expression levels of **(A)** colistin resistance-associated genes (*phoP* and *pmrD*), **(B)** efflux pump genes (*acrB*, *tolC*), and **(C)** global regulon (*ramA*) in the parent CR-hvKP strain, C1398, and its corresponding mutants. In the colistin-resistant mutants selected using colistin, we noted significant differences in the expression levels of *phoP* (*p* = 0.0005) and *pmrD* (*p* = 0.0016) in mutant C1398 C-2 when compared with those in the parent strain, while the transcription levels of *acrB* (*p* = 0.0037), *tolC* (*p* = 0.0001), and *ramA* (*p* = 0.0049) in C1398 C-1 were significantly different from those in the parental strain. In the tigecycline-selected mutants, the expression levels of *pmrD* (*p* = 0.0007 for C1398 T-1, *p* = 0.049 for C1398 T-2), *acrB* (*p* = 0.0021 for C1398 T-1, *p* = 0.0113 for C1398 T-2), *tolC* (*p* = 0.0003 for C1398 T-1, *P* < 0.0001 for C1398 T-2), and *ramA* (*p* = 0.0179 for C1398 T-1, *p* = 0.0276 for C1398 T-2) were significantly higher in the mutants when compared to the parent strain. *: *p* < 0.05, ^**^: *p* < 0.01, ^***^: *p* < 0.001.

Colistin resistance is primarily mediated by modification of the lipid A moiety in lipopolysaccharide by phosphoethanolamine (PEtN) and 4-amino-4-deoxy-L-arabinose (L-Ara4N) (Mmatli et al., 2020). The colistin MICs for the colistin-resistant mutants, C1398 C-1, C1398 C-2, and C1398 T-1, produced under either colistin or tigecycline pressure, did not change synchronously, suggesting differences in the evolutionary mechanisms of this resistance in these mutants (Figures 3A,B). The colistin-resistant mutants from the final day of selection were sequenced using Illumina HiSeq and Nanopore, and the SNPs and indels were analyzed to understand the evolutionary mechanisms used to achieve this resistance. We checked for mutations, insertions, and deletions in genes linked to colistin resistance, such as *crrB*, *mgrB*, *phoPQ*, *pmrABCD*, *pmrH*, and *pmrI*. For the colistin-resistant mutant C1398 T-1 selected under tigecycline pressure, a frameshift in the *lon* gene [1,286–1,295 nt deletion (CTTCCGACAT)] resulted in an increase in the tigecycline MIC (2 mg/L), whereas the colistin resistance (64 mg/L) was possibly due to a ∼4.4 kb deletion including the *mgrB* locus and several other genes, including the transcriptional regulator gene *kdgR*, *yobH*, *yebO*, *yobF*, cold shock protein gene *cspC*, cell division protein gene *ftsI*, and *rlmA* (Figures 4A,B). The expression of *pmrD* (*p* = 0.0007), *acrB* (*p* = 0.0021), *tolC* (*p* = 0.0003), and *ramA* (*p* = 0.0179) was significantly increased in C1398 T-1 compared with that in its parent strain (Figures 5A–C). The mutant C1398 C-2, selected under colistin pressure, presented with a deletion of a similar region (∼4.6 bp) containing *kdgR*, *yobH*, *mgrB*, *yebO*, *yobF*, *cspC*, *ftsI*, and *rlmA* (Figures 4A,C) and significantly higher *phoP* (*p* = 0.0005) and *pmrD* (*p* = 0.0016) expression than the parent strain C1398 (Figure 5A). Notably, a 4 bp (AAGA) or 5 bp (GGCAA) target site duplication was observed at the end of each deletion region, suggesting a recombination event that might have caused the deletion. Similar resistance mechanisms have been identified in an ST15 KPC-2-producing hypermucoviscous strain isolated from a blood culture of a 79-year-old patient in Brazil with a ∼1.3 kb deletion which was responsible for the colistin resistance, containing *mgrB*, *yebO*, *yobH*, and the transcriptional regulator *kdgR* (de Araújo Longo et al., 2021). A ∼5.4-kb deletion of the similar region was also detected in an ST2261 *K. pneumoniae* isolate colonizing in a healthy Swiss female after a trip to India, resulting in the MIC of polymyxins > 4 mg/L (Bernasconi et al., 2018).

However, no SNPs were found in the known colistin resistance genes in C1398 C-1. The sequences of *mgrB, crrB*, *phoPQ*, *pmrABCD*, *pmrH*, and *pmrI* did not exhibit any mutations. In addition, no significant change in the expression of the two-component system genes (*phoP*) or the downstream modification of lipopolysaccharide (*pmrD*) was observed, suggesting that this mutant relied on other mechanisms for increasing its resistance to colistin.

CCCP is a non-specific efflux pump inhibitor that abolishes proton motive force and energy source, affecting pump function (Li et al., 2015). A significant decrease in colistin MIC was described in C1398 C-1 when treated with CCCP (10 mg/L), decreasing from 16 to ≤ 0.5 mg/L, suggesting a potential role for this efflux pump in the colistin resistance of C1398 C-1. Upregulation of the AcrAB-TolC pump has been found to induce colistin resistance in *K. pneumoniae* (Naha et al., 2020), and both *acrB* (*p* = 0.0037) and *tolC* (*p* = 0.0001) experienced significant increases in expression in the C1398 C-1 strain (Figure 5B). Furthermore, we identified an additional frameshift mutation in *ramR* [207–213 nt deletion (AATGATC)] in the WGS data, although there were no changes in the sequences of *ramA*, *acrA*, *acrB*, *rpsJ*, or *soxS* (Table 1). We then used a PAβN inhibitory assay to confirm the role of the AcrAB-TolC pump in colistin resistance. PAβN was found to be a broad-spectrum inhibitor of the resistance-nodulation-cell division exporter (RND) efflux pumps, especially the AcrAB-TolC pump (Li et al., 2015). However, we found no changes in the MIC for colistin in this strain in the presence of PAβN, but the tigecycline MIC decreased, from 1 to 0.5 mg/L, when PAβN was added to C1398 C-1, suggesting that overexpression of the AcrAB-tolC pump resulting from the *ramR* mutation was responsible for the slight increase in the tigecycline MIC of this strain rather than its colistin resistance. Taken together, these results suggest that the AcrAB-TolC pump could be ruled out as the main reason for colistin resistance in these strains.

Other efflux pumps, including KpnEF, along with alterations in the LPS and outer membrane proteins mediated by changes in the *ramA* and *omp* genes have also been reported to decrease susceptibility to colistin (Srinivasan and Rajamohan, 2013; De Majumdar et al., 2015; Kádár et al., 2017). However, we found no mutations in KpnEF in our strains. We did find an increase in *ramA* (*p* = 0.0049) expression in C1398 C-1 (Figure 5C), but this seems to be linked to tigecycline and not colistin. Further studies investigating the role of *ramA* and other efflux pumps in conferring colistin resistance in C1398 C-1 are urgently needed. In addition, only one strain was investigated for the evolution of colistin resistance under tigecycline or colistin pressure, posing difficulties for comprehensive understanding in the colistin resistance in CR-hvKP. Therefore, we are in the process of recruiting more CR-hvKP strains for our evaluation of colistin evolution and hope to use these data to expand on the associations between colistin resistance and tigecycline pressure in CR-hvKP strains.

## Conclusion

In conclusion, we report a case of severe neonatal bacteremia caused by colistin-resistant KPC-2–producing ST11-K64 CR-hvKP. Our findings reveal a link between colistin resistance and tigecycline pressure in CR-hvKP strains and demonstrate that colistin-resistant CR-hvKP strains can develop rapidly in response to both tigecycline and colistin pressure using several different evolutionary mechanisms. This finding presents a critical issue for CR-hvKP infection control, particularly with the potential increased use of tigecycline and colistin in clinical treatment.

## Data Availability Statement

The datasets presented in this study can be found in online repositories. The names of the repository/repositories and accession number(s) can be found in the article/supplementary material.

## Author Contributions

HW conceived and designed the study. YZ and XW performed the experiments and analyzed the data. YZ wrote the manuscript. SW and QW collected the data. YZ, SS, HL, and HC collaborated in the bioinformatics analysis. All authors contributed to the article and approved the submitted version.

## Conflict of Interest

The authors declare that the research was conducted in the absence of any commercial or financial relationships that could be construed as a potential conflict of interest.

## Publisher’s Note

All claims expressed in this article are solely those of the authors and do not necessarily represent those of their affiliated organizations, or those of the publisher, the editors and the reviewers. Any product that may be evaluated in this article, or claim that may be made by its manufacturer, is not guaranteed or endorsed by the publisher.

## Footnotes

1
http://rast.nmpdr.org/

2
http://www.genomicepidemiology.org/

3
https://github.com/katholt/Kleborate
